# The Role of Traffic Volume on Sound Pressure Level Reduction before and during COVID-19 Lockdown Measures—A Case Study in Bochum, Germany

**DOI:** 10.3390/ijerph20065060

**Published:** 2023-03-13

**Authors:** Falk Hemker, Timo Haselhoff, Susanne Brunner, Bryce T. Lawrence, Katja Ickstadt, Susanne Moebus

**Affiliations:** 1 Institute for Urban Public Health (InUPH), University Hospital Essen, University Duisburg-Essen, 45147 Essen, Germany; 2 Department of Statistics, TU Dortmund University, Vogelpothsweg 87, 44227 Dortmund, Germany; 3 Research Group Landscape Ecology and Landscape Planning, Department of Spatial Planning, TU Dortmund University, Vogelpothsweg 87, 44227 Dortmund, Germany

**Keywords:** environmental noise, COVID-19, lockdown, acoustic environment, SPL, traffic noise, mixed-model regression, wind speed, rainfall

## Abstract

During the SARS-CoV-2 pandemic, sound pressure levels (SPL) decreased because of lockdown measures all over the world. This study aims to describe SPL changes over varying lockdown measure timeframes and estimate the role of traffic on SPL variations. To account for different COVID-19 lockdown measures, the timeframe during the pandemic was segmented into four phases. To analyze the association between a-weighted decibels (dB(A)) and lockdown phases relative to the pre-lockdown timeframe, we calculated a linear mixed model, using 36,710 h of recording time. Regression coefficients depicting SPL changes were compared, while the model was subsequently adjusted for wind speed, rainfall, and traffic volume. The relative adjusted reduction of during pandemic phases to pre-pandemic levels ranged from −0.99 dB(A) (CI: −1.45; −0.53) to −0.25 dB(A) (CI: −0.96; 0.46). After controlling for traffic volume, we observed little to no reduction (−0.16 dB(A) (CI: −0.77; 0.45)) and even an increase of 0.75 dB(A) (CI: 0.18; 1.31) during the different lockdown phases. These results showcase the major role of traffic regarding the observed reduction. The findings can be useful in assessing measures to decrease noise pollution for necessary future population-based prevention.

## 1. Introduction

The SARS-CoV-2 pandemic has been, and continues to be, a challenge with a huge impact on public life. However, its effects on the urban acoustic environment represent an unprecedented chance for researchers to examine the relationship between sound sources and sound pressure levels (SPL). These observations are important since noise is one of the major problems contributing to negative health outcomes in urban populations [1]. Already in 1996, the European commission estimated that around 20 million EU citizens suffer under noise levels deemed as unacceptable, and another 170 million EU citizens lived in areas where noise could cause serious annoyance during daytime [2]. This is especially true for urban environments, where noise levels are usually higher because of the density and proximity of living accommodations, traffic, construction sites, leisure noise, and many other sound sources. Higher noise levels are associated with cardiovascular diseases and metabolic effects [3], including arterial hypertension [4], sleep disturbances [5], effects on cognition and scores in national standardized tests [6], emotional and conduct disorders in childhood as well as hyperactivity symptoms in children [7], annoyance [8], and adverse birth effects [9]. One of the main contributors to noise is road traffic [10]. This is also true for subjective perception of noise sources. In a questionnaire conducted during the lockdown in Argentina from 14 April 2020 to 26 April 2020, participants rated traffic as the predominant noise source as well as the most annoying noise source before the lockdown [11]. The World Health Organization (WHO) recommends road traffic noise to not exceed L_den_ 53 a-weighted decibels (dB(A)) to reduce the probability of adverse health effects [1]. The European Environmental Agency (EEA) estimated that, in its member countries, 125 million people could be exposed to road traffic noise above L_den_ 55 dB(A), of which 37 million exceed an exposure of L_den_ 65 dB(A). The EEA computed that there are at least 10,000 premature deaths due to noise in Europe each year. A majority of which (89%) are mainly associated with road traffic noise [12]. However, the WHO recommendations for noise reduction are challenging to implement in crowded cities. To achieve traffic noise exposure reduction goals, it is necessary to gain knowledge about the needed change in road traffic that results in less noise exposure for urban populations.

In many parts of the world, the start of the first COVID-19 lockdown measures resulted in decreased SPLs. In Madrid, working days were around 4–6 dB(A), L_d_, L_e_, and, L_n_ quieter [13]. Overall there was a reduction of 6 to 7 dB(A) in Montreal [14], 5.4 L_Aeq_ in London [15], 4–6 dB(A) L_den_ in France [16], 8.77 dB(A) L_den_ in Rome, and 7.3 dB(A) L_den_ in Milan [17]. In addition, the WHO thresholds on noise exposure were less often exceeded in Dublin during the lockdown [18]. In quieter areas, effects are not as extensive. A study in Japan found only a slight reduction of up to 2 dB(A) and even a slight increase in SPLs [19]. It is notable that, in Japan, there was no strict lockdown at the beginning of the pandemic, but a ‘state of emergency’ in which the government used recommendations and did not enforce restrictions to slow down the spread of the disease. For the German city of Bochum, an earlier analysis showed that the five weeks preceding the first lockdown were 5.1 dB(A) louder than the first five weeks during the lockdown [20]. The general decrease is not only objectively measurable, but people all over the world reported sensing a quieter environment during the beginning of the pandemic [21].

Given the exceptional circumstances of the pandemic, the opportunity to measure SPL in an urban environment with drastically reduced traffic volume arises. While there are calculations available which can model the expected SPLs when reducing road traffic volume, such as those designed for the European noise assessment CNOSSOS-EU [22], there was rarely a possibility to gather actual data on such a long-lasting and drastic change. The challenge in answering the question of how much of the observed reduction can be attributed to traffic lies in the necessity to measure traffic and SPLs simultaneously during the lockdown(s). Many studies have either sound data measurements but limited traffic volume data [13,14], or measured traffic volume and modelled the SPL data [23]. One study in France measured dB(A) and showed that reduction in traffic volume seemed to behave in a similar pattern, but was missing a statistical model to connect these findings [16]. The same is true for measurements from three microphone stations and traffic data in a suburban area on a main street in Rome [17]. This shows the need for providing a model to test whether traffic volume is indeed the major contributor to the decrease in SPLs during the pandemic. This need for a quantitative model is also stated in other research [24]. Furthermore, by measuring the road traffic and SPLs before and during the pandemic, it is possible to gain an outlook on what sound pressure exposure is to be expected in the urban acoustic environment with a drastic change in road traffic volume. While the reduction in traffic volume during the pandemic was unprecedented, there are articles arguing that similar effects on SPLs can be achieved by combining other solutions besides issuing a public lockdown [16].

The aim of this study is to describe the development of SPLs over varying lockdown phases in the city of Bochum, Germany and to estimate the role of traffic volume regarding these changes. For this purpose, we analyzed longitudinal sound pressure data recorded at 23 urban locations in Bochum. A mixed model approach is used to examine the role of traffic volume on SPL changes during lockdown phases.

## 2. Materials and Methods

### 2.1. Sound Data

For this study, SPL measures from the SALVE (AcouStic QuAlity and HeaLth in Urban EnVironmEnts) project [25] were used to calculate dB(A) values using Kaleidoscope V. 5.4.2 [26]. The study was conducted in Bochum, Germany, part of the Ruhr Metropolitan Area, one of the most densely populated areas in Europe, with over 5 million inhabitants [27]. As part of the SALVE project, automated recordings were made starting on 06.05.2019 using 24 Wildlife Acoustics SM4 recorders [28] as stationary automated aural devices (AADs) [25,29]. We made 3-minute recordings every 26 min through 28.04.2021. As means to measure SPL, the built-in microphone of the SM4 device (Sensitivity: −35 ± 4 dB (0 dB = 1 V/pa @ 1 kHz); Signal to Noise Ratio: 80 dB typical at 1 kHz (1 Pa, A weighted network)) was used [28]. The SM4 were factory calibrated upon field placement and calibrated again after 12 months of continual use, then again 12 months later, using an MG 4010 Calibrator [30], which conforms with DIN EN60942-2003, Class 1. All devices were within the factory listed tolerance of ±4 dB(A), usually within 1 or 2 dB(A). The measured data were aggregated in dB(A) as mean per hour and we eliminated one of the 24 devices due to mechanical error (n_AAD_ = 23). The timeframe from 19 May 2020 to 14 June 2020 had to be excluded due to a pause in recording for device maintenance. In total, 734,192 3-min recordings, resulting in 36,710 h of audio data, were considered (Appendix A).

### 2.2. Traffic Data

Traffic volume data were obtained from the website of the federal office for road traffic (Bundesanstalt für Straßenwesen) [31] and the road construction office of North Rhine Westphalia (Landesbetrieb Straßenbau Nordrhein-Westfalen) on the Strassen.nrw website [32]. The data set comprises the number of vehicles driving on the A40 (Autobahn/highway 40). The A40 is one of Germany’s most used highways, connecting the cities of the Ruhr Metropolitan Area with each other. We used data from station No. 5113, which is the closest to the city of Bochum. These data were used as a proxy to estimate the traffic volume in Bochum. Corresponding to dB(A) data, we estimated mean counts of vehicles per hour. To ease interpretability of mixed model analysis, we calculated the binary logarithm (log_2_) of the vehicle count, so that an increase of one corresponds to a doubling of vehicle counts. For a map showing where the AADs and traffic counting station are located in Bochum, see Appendix B.

### 2.3. Lockdown Phases

To analyze the impact of lockdown measures on mobility and SPL, we defined two timelines as observation periods. We defined a pre-pandemic reference phase from 6 May 2019 to 15 March 2020, followed by the during-pandemic timeframe from 16 March 2020 to 28 April 2021. To account for the different degrees of austerity of the lockdown measures, we further divided the during-pandemic timeframe into four lockdown phases, including ‘first lockdown’, ‘opening period’, ‘lockdown light’, and ‘second lockdown’. The first lockdown began on 16 March 2020, since travel restrictions, school, public entertainment places, and retail store closures were enacted in Germany on this date and the following days [33,34,35]. On 4 May 2020, the opening period began, where hairdressers, museums, and zoos were allowed to open their gates, followed by loosening of restrictions on retail stores and meeting in public spaces [36]. Soon after, schools were gradually reopened [37]. Rising SARS-CoV-2 incidences during autumn resulted in the lockdown light on 2 November 2020. Restaurants, bars, and clubs had to close, more severe restrictions on public meetings were enacted, and stores had to reduce the number of customers that were allowed inside [38]. Schools and stores remained open. Not yielding the desired result in greatly slowing down the rise of SARS-CoV-2 incidence, further lockdown measures were enacted on 16 December 2020. Thus, the second lockdown phase had begun, with the earlier partial closure of schools, retail stores, hairdressers, and other services [39,40], in addition to private gathering restrictions (with a small easing of restrictions from the 24–26 December 2020 for celebrating Christmas). Selling of fireworks was banned for New Year’s Eve. For an overview on major events and measures in the city of Bochum, refer to Appendix C.

### 2.4. Confounders

The main quantity that determined which measures were in place during the analysis timeframe was the incidence rate of SARS-CoV-2 infections. Infection rates during the pandemic were found to be very closely associated with seasonality [41,42]. To account for seasonal influences on SPLs, we considered wind speed and rainfall. Wind speed and rainfall are two strong geophonic quantities impacting dB(A) and show a seasonal pattern [43]. Data on wind speed and rain were obtained via the Ruhr University Bochum’s (RUB) weather station [44]. These data were used as a proxy to estimate the confounding effects of wind speed and rain on dB(A) for all recording places. Wind speed was measured as meters per second (m/s) and rain as precipitation in millimeter. Both were aggregated as mean per hour for every individual day.

### 2.5. Statistical Methods

For the descriptive analysis, we calculated means and standard deviations per hour from every 697 days. Means were calculated regarding the logarithmic scale of sound pressure levels measured in decibel.

A-weighted decibels and traffic volume were separated in the five defined phases. To visualize the course of all five phases over the observed period, we used means per day to avoid overplotting.

As there are many recordings for each of the 23 AADs placed within different types of urban environments, a two-level hierarchical structure for the recordings was created, with the assumption that recordings within one AAD tend to be more alike than between AADs. Because of this data structure, a mixed model approach [45,46,47], incorporating fixed and random effects, was used to analyze the association between dB(A) and lockdown phases relative to the pre-lockdown timeframe. Due to the different environments of the AADs, we assumed different effects from the lockdown phases and weather on each recording device.

First, a crude model, Model I, was analyzed, including only an intercept and the fixed and random effects for all four lockdown phases, x_1it_,

y_it_ = 𝛽_0_ + 𝛽_1_x_1it_ + u_0i_ + u_1i_ (x_1it_) + 𝜖_it_.

Here, i = 1,…, 23, is used as an index for ADD devices, u_0i_ denotes the random intercept per ADD, and u_1i_ the random slope per ADD. 𝜖_it_ is an overall error term with t = 1, …, 16,595, denoting individual hours over the measured timeframe per device.

In a second step, we additionally consider wind speed and rainfall (Appendix D), x_2it_ and x_3it_, respectively, as fixed and random effects in order to adjust for the seasonal effects of the lockdown phases. In the following, this model will be referred to as Model II:

y_it_ = 𝛽_0_ + 𝛽_1_x_1it_ + 𝛽_2_x_2it_ + 𝛽_3_x_3it_ + u_0i_ + u_1i_ (x_1it_) + u_2i_ (x_2it_) + u_3i_ (x_3it_) + 𝜖_it_.

To answer the question on how far the observed change in dB(A) can be explained by traffic volume, Model III was considered. Here, traffic volume, x_4it_, was added as a fixed effect with a random slope u_4i_.

y_it_ = 𝛽_0_ + 𝛽_1_x_1it_ + 𝛽_2_x_2it_ + 𝛽_3_x_3it_ + 𝛽_4_x_4it_ + u_0i_ + u_1i_ (x_1it_) + u_2i_ (x_2it_) + u_3i_ (x_3it_) + u_4i_ (x_4it_) + 𝜖_it_.

All analyses were performed using R version 4.1.3 [48], package ‘glmmTMB’ version 1.1.3 [49] for regression analysis and ‘seewave’ version 2.2.0 for averaging dB(A) values and calculating standard deviations [50].

## 3. Results

In this chapter, the descriptive analysis of the sound data and traffic data, as well the results of the mixed model analysis, are presented.

### 3.1. Changes in dB(A)

**Figure 1:**
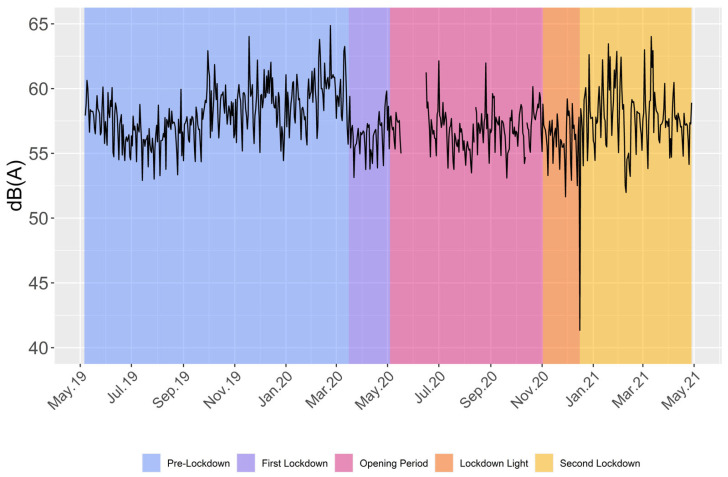
Mean daily dB(A) levels over the study period. Fluctuations arise from differences between days. The missing data between May and June 2020 is due to maintenance of the recording devices.

Findings show that mean dB(A) decreased during the first lockdown compared to the pre-lockdown (Table 1). A notably sharp decrease in dB(A) following the beginning of the first lockdown can be seen in Figure 1. As expected, mean dB(A) increased during the opening period while showing a similar pattern to pre-pandemic values with a slight decrease. The lockdown light showed the overall lowest observed mean of 56.36 dB(A). Counterintuitively, mean dB(A) increased—despite more stringent measures—during the second lockdown.

### 3.2. Traffic Volume on A40

**Figure 2:**
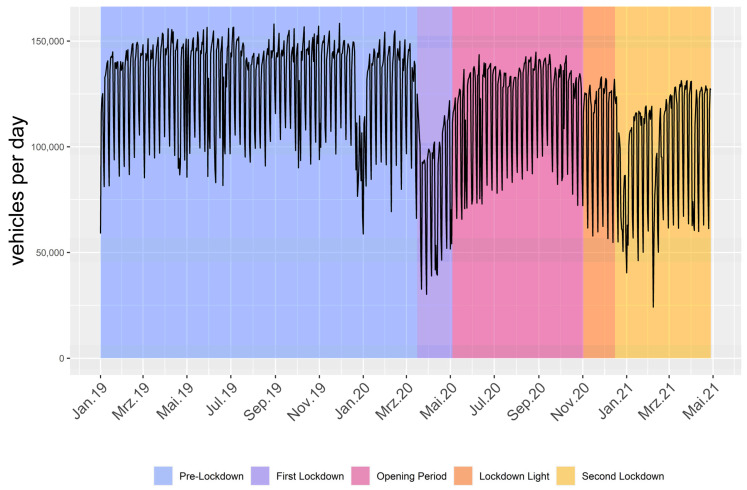
Traffic volume per day over the study period on the A40 between the German cities of Bochum and Essen.

A weekly pattern can be seen in the traffic volume data, with more volume on the A40 during weekdays than weekends. The results of enforced lockdown measures on traffic volume during the pandemic compared to the pre-pandemic phase are clearly visible (Figure 2). After an initial strong drop during the first lockdown (3520.4 vehicles/h), traffic volume gradually rose during the opening period (4996.5 vehicles/h) until being reduced again (4599.6 vehicles/h), followed by another subsequent reduction during the second lockdown (4119.3 vehicles/h). It is notable that, around Christmas and New Year, the road traffic usually declines, which overlaps with the second lockdown. Still, there was an overall lower traffic volume at the end of December 2020 than in 2019.

### 3.3. Mixed Model Analysis

We compared the crude Model I with Model II, which was adjusted for wind speed and rainfall, before finally adjusting for traffic volume in Model III with respect to the fixed and random effects coefficient estimation of the different lockdown phases. Table 2 depicts the effect estimates of all models and their respective 95% CI intervals.

The feasibility of the mixed model approach is supported by the observation that adding a random intercept to the fixed-effects-only-model containing just the lockdown phase results in a significant change in deviance, indicating a higher model fit (χ^2^(1) = 261,622, *p* < 0.01). The random coefficients standard deviation for the intercept across AADs was greatest, showing variation in intercepts of SPLs for AADs, and thus underlining the hierarchical structure of the data. The random slopes for lockdown phases showcase variation in the impact of responses to lockdown measures on individual AADs.

When adding rainfall and wind speed for Model II, the calculated reduction diminishes. While the first lockdown still shows the greatest reduction, it is now followed by the second lockdown, which previously depicted the lowest reduction unadjusted for weather phenomena. The opening period and lockdown light show only a slight reduction in SPL. No substantial change is found between the random effect estimates. In Model III, traffic volume was added to analyze how much of the found change in dB(A) can be attributed to it. Traffic data were logarithmized using a base of two, resulting in a more linear relation of traffic volume and SPLs in dB(A). Model III shows positive effect estimates for all phases except for the opening period, with a weak negative effect size (Table 2). However, the confidence intervals indicate the possibility of no relative change in most phases. The estimate of traffic volume reveals an increase of 2.34 dB(A) when doubling traffic volume (increasing the coefficient of traffic volume by one). The random slope of traffic volume with 0.86 (0.64, 1.15) showcases a heterogenous impact of changed vehicle traffic volume on dB(A) depending on the location of the observed AAD monitoring site.

## 4. Discussion

The objective of this paper was to describe the reduction in SPLs over the course of the different lockdown phases of the COVID19 pandemic between March 2020 and April 2021 and to analyze the role traffic played regarding these reductions, while adjusting for the confounders wind speed and rainfall.

### 4.1. Sound Pressure Levels Compared between Pre-Pandemic and Different Lockdown Phases

Our descriptive results show a substantial reduction in SPLs during the pandemic lockdown measures as compared to pre-pandemic conditions. These reductions differ over the course of lockdown phases. The patterns slightly change when controlling for the hierarchical data structure as well as for wind speed and rainfall. Before adjustments, the lockdown light depicts the greatest reduction and the second lockdown the least. The lower dB(A) from July to August (see Figure 1) can probably be explained by school holidays. The low levels during the second lockdown can be explained by diminished public events and mobility during the winter holiday and the banning of fireworks and gatherings for New Year’s Eve compared with 2019. After adjustments, the lockdown patterns change between Model I and Model II. This means that the sound pressure reduction was partially associated with weather phenomena. This highlights the necessity of adjusting for weather phenomena in longitudinal sound data. This is consistent with the statement Čurović et al. made, analyzing noise levels during the pandemic at a port, stating that wind speed should be considered in long-term measurements [51]. The first lockdown now shows the greatest reduction while being the one with the strictest measures (e.g., only being allowed to privately meet with one other person with only a few exceptions [52]). Estimates of the second lockdown depict the second strongest reduction in Model II. Besides the slightly loosened measures compared to the first lockdown, a change in public behavior resulting from adaption after the initial impact of restrictions in the first lockdown could play a role in the lesser reduction during the second lockdown. This pattern can also be seen in the traffic data. It might be further amplified by the start of vaccinations against SARS-CoV-2 in December 2020 [53]. Model II estimates a decrease in sound pressure, ranging from −0.25 to −0.99 dB(A), which is not as large as the reduction found in other studies, mostly lying around 4 to 8 dB(A). One main difference between the mentioned studies and the present research is the observed timeframe, with our study analyzing the longest period as well as adjusting for weather phenomena. As already mentioned, the unadjusted initial decrease in Bochum of 5.1 dB(A) [20] is comparable to international research results. Figure 1 shows the pattern of strongly reduced SPLs after the start of a new lockdown. It is plausible that the longer the measures were in place, the more public behavior (and thus SPLs) normalized. These circumstances could explain the smaller reduction in this study since it observes more than just the beginning of a lockdown phase. This trend of regressing towards standard levels following an initial decrease is supported by similar findings in a Spanish study [54]. Overall, estimates of Model II full-on lockdowns (‘First’ and ‘Second Lockdown’) indicate an association of reduction in SPLs and responses to political measures.

### 4.2. The Role of Traffic in Sound Pressure Reduction during the Pandemic

According to Model II, a reduction in SPL can be observed, with the greatest reduction found during the first lockdown. To analyze the role of traffic regarding these changes, logarithmized traffic volume was added in Model III. The observed decrease in traffic volume is in line with reports all over the world [13,23,55]. Comparing Model II and III, it becomes obvious that most of the measured reduction during the pandemic can be associated with changed traffic volume in the urban environment. This is displayed by the fact that there still is a reduction (negative estimates for phases) when only adjusting for wind speed and rain, while there is close to none, or even an increase, when controlling for traffic volume. After adjusting for traffic volume, the decrease in sound pressure level for the first lockdown diminishes, and even an increase can be observed. The estimates of the remaining phases show a similar, yet smaller, change in the same direction. Especially the opening period is comparable, with no change, which suggests an overall low impact of pandemic circumstances during this phase. The reversing of the other three estimates indicates that changes in traffic volume during the pandemic can explain SPL reduction during the pandemic. This previously suspected relation [10,18] is statistically affirmed in this study.

While most studies claim that traffic is (one of) the main contributor(s) to SPL change [13,14,23,51], there are some findings highlighting the importance of location and potential other factors [15]. In Madrid, reduction during the weekend is especially visible in traffic dominated areas. However, at the same time, the greatest reduction is found at the Plaza del Carmen which is less influenced by traffic than other urban locations in Madrid [13]. In Girona, Spain, reduction of traffic noise in places shaped by urban life show a greater reduction than those heavily influenced by traffic [56]. Adding to this, positive SPL estimates in Model III indicate presently not included factors on SPL in the urban environment. These unobserved factors have a comparable effect in increasing SPLs as traffic has in decreasing them. The already mentioned study in Madrid found less SPL reduction than expected given the drastic decrease in traffic of 85% [13]. As a possible reason, higher vehicle speed on emptier roads and sound traveling from busy roads to quieter places without the interference of road traffic noise are discussed. A study at a Brazilian hospital also highlights the possible influence of increased vehicle speed [57]. These mechanisms potentially contribute to the estimation in Model III. Possibly, an increase in other means of transportation, such as walking or taking a bike, given a changed public behavior, could also contribute to the estimation. Since the exceptional circumstances during the pandemic had many different impacts on society, other factors than mobility, such as social events, cultural aspects, (recreational) sports, leisure activities, and a change in working routines could also play a role. Thus, further research is needed to ascertain how such factors cause an increase in SPL.

### 4.3. Strength and Limitations

In this study, 23 automated aural devices were used to capture the SPLs before and during the SARS-CoV-2 pandemic. This provides a high resolution in temporal data. The timeframe in which sound was measured makes it possible to examine the direct effect of the pandemic and political measures on the urban SPLs. Additionally, traffic volume data were obtained in a high temporal resolution, giving the opportunity to calculate a model on dB(A) and car traffic volume that is based on a radically changed urban environment due to mitigation measures. The inclusion of wind speed and rainfall allowed for controlling for (seasonal) weather effects.

Besides these strengths, some limitations deserve special mention. An even longer observation period would be of great value, especially for the non-pandemic period. This would make it achievable to compare day by day values between the years and to better control for seasonal impacts. Furthermore, in this study, most microphones were placed at least in the proximity of roads where traffic should be a main contributor to noise levels. In other studies which analyzed open public places, in which other human activity has a stronger influence (e.g., locations of festivals, great sport events, touristic activity, or marketplaces), the decrease is most likely due to changes in these aspects [58]. Spatial resolution of the data is limited; 23 AADs cannot cover all the different areas in a city with over 350,000 citizens. In future studies, it would be beneficial to have a higher number of devices deployed in the field, even though, considering the long timeframe that was investigated, this will pose a challenge. The limited spatial resolution is also relevant regarding the traffic data, as only one counting station was used. This means that the data obtained from the A40 can only function as a proxy for the actual road traffic inside the city. While this study focused on the outside urban environment, for many people, inside noise levels are another important parameter which should be addressed in future research. Lastly, these data are limited to the city of Bochum. Further research would have to be conducted in other cities to examine whether the role of traffic regarding the reduction in sound SPLs is comparable and where differences are to be found.

## 5. Conclusions

This study showcases the overwhelming influence of car traffic on sound pressure level reduction during the COVID-19 pandemic between March 2020 and April 2021, using a linear mixed model approach controlling for wind speed and rain. It denotes the data-based impact of reduced vehicle volume in Bochum on changed SPLs during the SARS-CoV-2 pandemic. The data show what changes could be achievable regarding a city’s SPLs when addressing road traffic as a major source of noise. During the lockdown, people were able to experience first-hand what an urban environment sounds like when vehicle traffic volume is substantially decreased. However, SPLs tend to regress to their pre-pandemic levels over time. This highlights the need for action if we want to keep the more pleasant and quieter (urban) environment, which was reported all over the world during the lockdown periods. While there is an ongoing effort to change means of transportation to reduce (private) vehicle traffic volume in urban environments, there are trends to return to the pre-pandemic status of high, traffic dominated SPLs in cities. This work adds that a political framework is effective in achieving the needed change. The positive coefficients for lockdown phases after adjusting for vehicle traffic volume on Model III indicate unidentified components influencing SPLs as large as traffic. Determining these parameters in future research and implementing the addressed political framework will contribute to an enduring improvement on quality of life for people living in the urban environment.

## Figures and Tables

**Table 1:** Mean values (M) and standard deviations (SD) of dB(A) and vehicles on A40 per hour for all devices are depicted over the four different lockdown phases and the pre-lockdown phase.

Phase	Period		dB(A)/hour		Vehicles on A40/hour	
	Start	End	M	SD	M	SD
Pre-Lockdown	5 June 2019	15 March 2020	58.33	4.5	5453.6	3243.4
First Lockdown	16 March 2020	3 May 2020	56.54	4.1	3520.4	2575.2
Opening Period	4 May 2020	1 November 2020	57.00	4.0	4996.5	3123.9
Lockdown Light	2 November 2020	15 December 2020	56.36	4.5	4599.6	3156.3
Second Lockdown	16 December 2020	28 April 2021	58.07	5.0	4119.3	2905.2

**Table 2:** Results of the linear mixed model analyses. Depicted are the crude and adjusted effect estimates with their corresponding 95% confidence intervals in brackets. Model I: unadjusted (crude), Model II: adjusted for wind speed (in m/s) and rainfall (in mm), Model III: additionally adjusting for traffic volume (log_2_(vehicles/h)). Lower number of observations for Models II and III are explained by some missing hours for wind speed and rain data.

	Model I	Model II	Model III
**Fixed Effects**			
(Intercept)	53.85 (51.32; 56.39)	49.37 (46.4; 52.35)	22.21 (19.21; 25.21)
First Lockdown	−1.54 (−1.99; −1.09)	−0.99 (−1.45; −0.53)	0.75 (0.18; 1.31)
Opening Period	−1.05 (−1.66; −0.44)	−0.35 (−0.94; 0.25)	−0.16 (−0.77; 0.45)
Lockdown Light	−0.90 (−1.62; −0.18)	−0.25 (−0.96; 0.46)	0.54 (−0.23; 1.3)
Second Lockdown	−0.72 (−1.35; −0.1)	−0.54 (−1.18; 0.09)	0.66 (−0.04; 1.36)
Wind Speed		1.32 (1.16; 1.49)	1.04 (0.85; 1.22)
Rainfall		1.89 (1.61; 2.16)	1.92 (1.65; 2.19)
Traffic Volume			2.34 (1.99; 2.70)
**Random Effects STD**			
(Intercept)	6.20 (4.64; 8.28)	7.27 (5.45; 9.71)	7.33 (5.49; 9.80)
First Lockdown	1.07 (0.79; 1.44)	1.11 (0.83; 1.5)	1.37 (1.02; 1.83)
Opening Period	1.49 (1.11; 1.99)	1.45 (1.08; 1.93)	1.49 (1.12; 1.99)
Lockdown Light	1.74 (1.3; 2.34)	1.71 (1.28; 2.29)	1.85 (1.38; 2.47)
Second Lockdown	1.52 (1.14; 2.04)	1.55 (1.16; 2.07)	1.71 (1.28; 2.29)
Wind Speed		0.40 (0.30; 0.54)	0.45 (0.34; 0.61)
Rainfall		0.66 (0.49; 0.89)	0.66 (0.49; 0.88)
Traffic Volume			0.86 (0.64; 1.15)
AIC	2,261,289	2,188,099	2,025,427
Num. obs.	352,455	351,144	351,144
Num. groups	23	23	23

**Figure A1:**
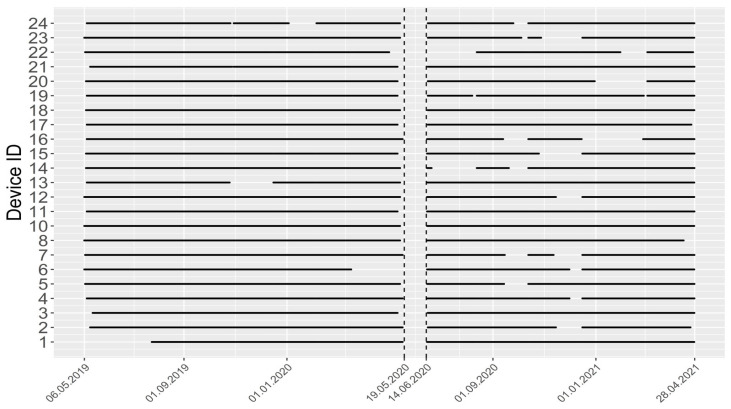
Depicted are the AADs, by their ID, over time on when there are recordings available. Breaks in a line represent a period of missing data. The vertical dotted lines show the maintenance timeframe lasting from 19 May 2020 to 14 June 2020. AAD 9 was excluded from every analysis because of a malfunction.

**Figure A2:**
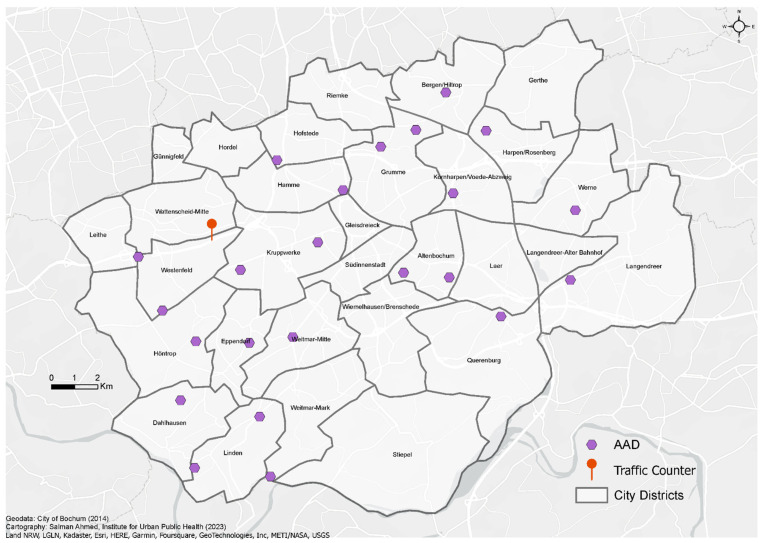
City districts with the depicted locations of AADs and traffic counting station No. 5113 in the city of Bochum, Germany.

**Figure A3:**
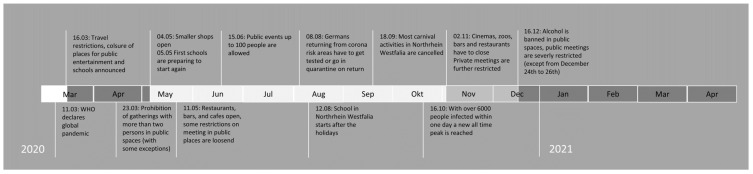
Overview of important measures and announcements, which affected the public life in Bochum. Lockdown phases were separated into ‘first lockdown’ (16 March 2020–3 May 2020), ‘opening period’ (4 May 2020–1 November 2020), ‘lockdown light’ (2 November 2020–15 December 2020), and ‘second lockdown’ (16 December 2020–28 April 2021). Full-on lockdowns are depicted dark grey, the lockdown light in a light grey, opening period in a bright grey, and pre-lockdown times in white. For detailed measures and references for each date see: 11.03 [59]; 16.03 [33,34,35]; 23.03 [52]; 04.05 [36]; 05.05 [37]; 11.05 [60]; 15.06 [61]; 08.08 [62]; 12.08 [63]; 18.09 [64]; 16.10 [65]; 02.11 [38]; 16.12 [39].

**Figure A4:**
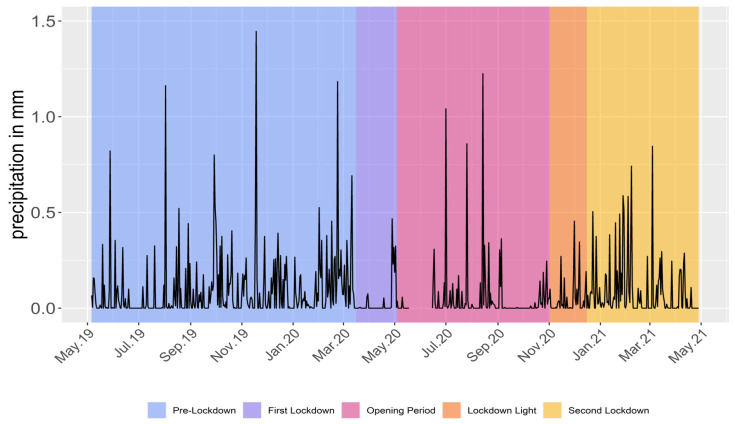
Daily means for precipitation in mm over time. The missing parts from mid-May to mid-June 2020 are attributed to the SALVE devices not being in the field because of maintenance. For consistency, the precipitation data are also not depicted in this timeframe.

**Figure A5:**
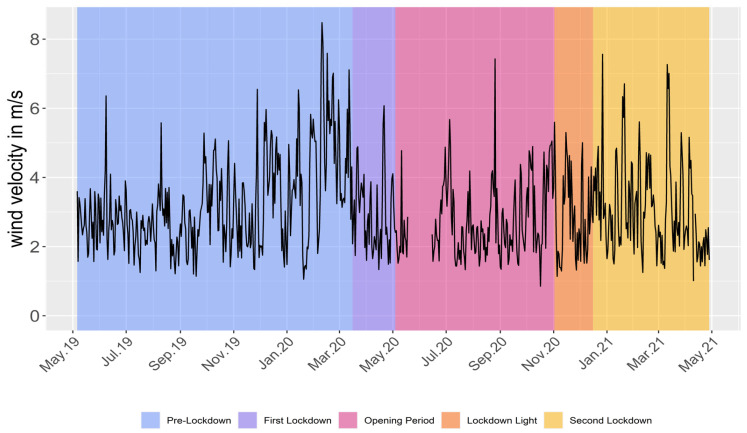
Daily means for wind in m/s over time. The missing parts from mid-May to mid-June 2020 are attributed to the devices not being in the field because of maintenance. For consistency, the wind data are also not depicted in this timeframe.

## Data Availability

The sound pressure level data presented in this study are available on request from the corresponding author. The data are not publicly available due to potential privacy issues. Third party data was used for weather data (available upon request with the permission of the Ruhr University Bochum, https://www.geographie.ruhr-uni-bochum.de/klima/rgs-weather.html.de) and vehicle data (available online: https://www.bast.de/DE/Verkehrstech-nik/Fachthemen/v2verkehrszaehlung/zaehl_node.html).

## Appendix A

Details on available data and timeframe.

## Appendix B

Map on AAD and traffic counting station location.

## Appendix C

Lockdown measures in Germany from 16 March 2020 to 28 April 2021.

## Appendix D

Wind and rain data.
